# Appearance-ideal internalization and appearance-related physical activity avoidance among female university students in Sichuan, China: the role of social physique anxiety and body surveillance

**DOI:** 10.3389/fpsyg.2026.1870099

**Published:** 2026-09-08

**Authors:** Yang Xu, Meilu Zhang, Xianlin Xiao, Huangyan Li

**Affiliations:** 1 Civil Aviation Flight University of China, Guanghan, China; 2 School of Humanities Education and Application, Sichuan Technology and Business University, Sichuan, China; 3 Faculty of Management, Shinawatra University, Pathum Thani, Thailand

**Keywords:** appearance-ideal internalization, appearance-related avoidance of physical activity and sport, body surveillance, female university students, muscular/athletic internalization, social physique anxiety, thin/low body fat internalization

## Abstract

Physical activity and sport can become appearance-evaluative settings when the body is visible, compared, or judged. This cross-sectional study examined whether thin/low body fat internalization (TLB) and muscular/athletic internalization (MA) were associated with appearance-related avoidance of physical activity and sport (APAS) in a convenience sample of 1,687 female university students recruited from 16 universities in Sichuan Province, China. Social physique anxiety (SPA) and body surveillance (BS) were examined as parallel intermediate variables. The primary latent-variable model was estimated with robust maximum likelihood, allowed TLB and MA to covary, and allowed the residuals of SPA and BS to covary. Bias-corrected confidence intervals for indirect associations were obtained from 5,000 bootstrap resamples. The primary model yielded robust χ^2^(550) = 600.111, CFI = 0.998, TLI = 0.998, RMSEA = 0.007, 90% CI [0.000, 0.011], and SRMR = 0.017. TLB and MA each showed positive direct associations with APAS (standardized β = 0.198 and 0.160), SPA (0.231 and 0.219), and BS (0.289 and 0.228); SPA and BS were also positively associated with APAS (0.193 and 0.207), all *p* < 0.001. Four specific indirect associations were statistically significant: TLB-SPA-APAS, B = 0.042, 95% bias-corrected CI [0.028, 0.061]; TLB-BS-APAS, B = 0.057, CI [0.039, 0.077]; MA-SPA-APAS, B = 0.050, CI [0.032, 0.073]; and MA-BS-APAS, B = 0.056, CI [0.037, 0.079]. Bootstrap contrasts did not indicate unequal contributions from SPA and BS. Directionally specified serial alternatives were statistically equivalent in fit and did not identify temporal ordering. Targeted sensitivity analyses supported the principal pattern across covariate, subgroup, university-cluster, item, and ordinal-estimation checks. Higher APAS scores were concurrently associated with lower self-reported activity frequency and shorter session duration. The findings describe positive direct and specific indirect associations in this Sichuan university sample; they do not establish causal or temporal processes.

## 1 Introduction

Physical activity supports physical and mental health, yet participation is shaped by more than available time, health knowledge, or general motivation. Global estimates indicate that a substantial proportion of adults do not meet recommended activity levels (Strain et al., 2024; World Health Organization, 2024). Among university students, movement habits develop within academic, social, and institutional environments that can either support or constrain participation. Research among Chinese female university students has linked physical activity with sedentary time and physical fitness, illustrating the relevance of this population to physical activity research (Guo et al., 2022). General inactivity measures, however, do not isolate avoidance motivated by body-, weight-, or appearance-related concerns.

The Tendency to Avoid Physical Activity and Sport Scale (TAPAS) was developed to assess a specific self-reported tendency to avoid physical activity and sport in response to weight stigma and appearance-related concerns (Bevan et al., 2022). Its construct is narrower than physical inactivity. A student may report appearance-related avoidance while still participating because of course requirements, health goals, social support, or established routines; another student may be inactive for reasons unrelated to appearance. Exercise settings may become body-evaluative when they involve fitted clothing, visible movement, sweating, public performance, mirrors, or peer comparison (Sabiston et al., 2014). The present study therefore focuses on appearance-related avoidance of physical activity and sport (APAS), while treating self-reported activity frequency and session duration as related but distinct behavioral indicators.

Sociocultural theories propose that appearance ideals become psychologically consequential when external standards are accepted as personally relevant criteria for self-evaluation. The Sociocultural Attitudes Towards Appearance Questionnaire-4 (SATAQ-4) distinguishes thin/low body fat internalization from muscular/athletic internalization (Schaefer et al., 2015). Thin/low body fat internalization emphasizes leanness and low body fat. Muscular/athletic internalization emphasizes a toned, firm, fit, or athletic-looking body. These ideals may overlap, yet they are not interchangeable. Prospectively, thin-ideal internalization predicted changes in body dissatisfaction, dieting, and compulsive exercise, whereas athletic-ideal internalization predicted change only in compulsive exercise (Homan, 2010). Experimental exposure to athletic-ideal imagery increased body dissatisfaction but did not increase exercise engagement relative to thin-ideal imagery (Robinson et al., 2017). Appearance-based exercise motivation also appears capable of coexisting with frequent exercise and less positive body image (Homan and Tylka, 2014). A muscular or athletic ideal should therefore not be treated as uniformly protective or uniformly harmful. When endorsed as an appearance standard, it may support approach motives while also making exercise settings feel evaluative when a perceived discrepancy from the ideal is salient.

The cultural setting requires a similarly balanced account. Appearance research developed largely in Western contexts cannot simply be transferred to China by citing translated instruments. A meta-analysis found that greater social-networking-site use was associated with stronger thin-ideal internalization among females, with appearance-related use showing a stronger association than broad use (Mingoia et al., 2017). A prospective study of young Chinese women found that appearance pressures and preferences associated with Chinese and other Asian media were more salient for several body-image concerns than complementary Western-media influences (Jackson et al., 2020). Research on Chinese social media has documented both persistent appearance-focused content and emerging body-positive messages that challenge appearance anxiety (Lang et al., 2023). Qualitative evidence also indicates that young women in China negotiate thinness, fitness, femininity, health, and body positivity within a changing media environment rather than adopting a single imported ideal (Lang and Ye, 2024). Longitudinal evidence from China further links social-media ideal exposure with later thin-ideal internalization and social appearance anxiety (Yao et al., 2024). These findings support a culturally situated investigation of appearance ideals without assuming that Chinese and Western women differ in a single uniform direction. The present sample does not provide a direct cross-cultural comparison.

Two body-related constructs may help clarify the simultaneous association between appearance-ideal internalization and APAS. Social physique anxiety (SPA) concerns affective apprehension that other people may evaluate one’s physique (Hart et al., 1989; Sabiston et al., 2014). Body surveillance (BS) concerns habitual monitoring of the body from an observer’s perspective, a central construct in objectification theory (Fredrickson and Roberts, 1997; McKinley and Hyde, 1996; Moradi and Huang, 2008). SPA is primarily interpersonal and evaluative; BS is primarily attentional and self-objectifying. Both have been connected with physical activity experiences. SPA has been associated with exercise motives, attitudes, and participation (Crawford and Eklund, 1994; Portman et al., 2018). Daily-life research has linked BS with less favorable affective judgments of physical activity (Gilchrist et al., 2021), and other work has connected BS with reduced intrinsic motivation for physical activity (Cox et al., 2019).

Evidence exists for several component relationships, but a narrower gap remains. Available studies do not clearly establish how two distinct appearance-ideal internalization dimensions relate to the TAPAS-defined avoidance tendency when SPA and BS are estimated simultaneously in a latent-variable model among female university students in China. The model also raises an unresolved ordering question: BS could be associated with heightened SPA, SPA could reinforce monitoring, or the two could co-develop. Cross-sectional data cannot adjudicate these possibilities. The primary aim was therefore to estimate simultaneous direct and specific parallel indirect associations, while testing directionally specified serial models only as exploratory competitors. The study also examined whether the principal results were stable across relevant background characteristics, university clustering, TAPAS item 10, and ordinal estimation, and whether APAS was concurrently associated with self-reported activity frequency and duration.

## 2 Literature review and hypotheses

### 2.1 Appearance-ideal internalization and APAS

Thin/low body fat internalization and muscular/athletic internalization represent related standards with different content. Thinness-oriented standards foreground body size, leanness, and fatness. Athletic-oriented standards foreground tone, firmness, visible fitness, and an appearance of bodily competence. Both can make exercise appearance-relevant, but their implications are ambivalent. Thin-ideal internalization has prospective links with body dissatisfaction, dieting, and compulsive exercise (Homan, 2010). Evidence concerning athletic-ideal internalization is more mixed: it prospectively predicted change in compulsive exercise but not body dissatisfaction or dieting (Homan, 2010). Experimental exposure to athletic-ideal imagery increased body dissatisfaction without increasing exercise engagement relative to thin-ideal imagery (Robinson et al., 2017).

APAS concerns avoidance for body-, weight-, or appearance-related reasons, not general exercise frequency. A positive association with APAS can therefore coexist with exercise participation. Weight stigma and internalized appearance attitudes have been linked with exercise avoidance (Vartanian and Novak, 2011), and the original TAPAS validation connected higher avoidance scores with lower enjoyment and participation (Bevan et al., 2022). Direct evidence involving both SATAQ-4 internalization dimensions and TAPAS remains limited, so the following hypotheses are theoretically derived extensions. To align the hypotheses with the simultaneous structural model, H1a and H1b refer to conditional direct associations after the other predictor and both intermediate variables are included.

*H1a*: TLB will show a positive conditional direct association with APAS in the simultaneous model.

*H1b*: MA will show a positive conditional direct association with APAS in the simultaneous model.

### 2.2 Specific indirect associations involving social physique anxiety

SPA arises when a person anticipates that others may evaluate the appearance of the body (Hart et al., 1989). Exercise contexts can make this concern salient through visibility, clothing, movement, and comparison (Sabiston et al., 2014). Internalized thinness standards may heighten concern that body size or body fat will be judged, while internalized athletic standards may heighten concern that the body does not look sufficiently toned or fit. Research has linked SPA with exercise attitudes and activity behavior (Crawford and Eklund, 1994; Portman et al., 2018), providing empirical support for its association with exercise-related experiences.

The cross-sectional design cannot establish that internalization temporally precedes SPA or that SPA temporally precedes APAS. The hypotheses therefore concern specific indirect associations within the specified covariance model.

*H2a*: TLB will show a positive specific indirect association with APAS involving SPA in the parallel model.

*H2b*: MA will show a positive specific indirect association with APAS involving SPA in the parallel model.

### 2.3 Specific indirect associations involving body surveillance

BS reflects recurrent monitoring of how the body appears from an observer’s perspective (Fredrickson and Roberts, 1997; McKinley and Hyde, 1996). Internalized appearance ideals can make visual self-monitoring more salient because the body is repeatedly assessed against thinness, tone, or fitness standards. Prior work has identified BS in associations between thin-ideal internalization and negative body-related experiences (Fitzsimmons-Craft et al., 2012). Within physical activity contexts, higher BS has been associated with less favorable activity-related affect and lower intrinsic motivation (Cox et al., 2019; Gilchrist et al., 2021). A person focused on how the body looks during movement may experience less attention to enjoyment, skill, or bodily function.

*H3a*: TLB will show a positive specific indirect association with APAS involving BS in the parallel model.

*H3b*: MA will show a positive specific indirect association with APAS involving BS in the parallel model.

### 2.4 Primary parallel model and exploratory serial alternatives

The primary model treated TLB and MA as correlated predictors and SPA and BS as simultaneous intermediate variables. Direct paths from both internalization dimensions to APAS were retained. The residuals of SPA and BS were allowed to covary because the two constructs may share unmeasured contemporaneous influences after their associations with TLB and MA are considered. No hypothesis was assigned to a temporal order between SPA and BS.

**Figure 1:**
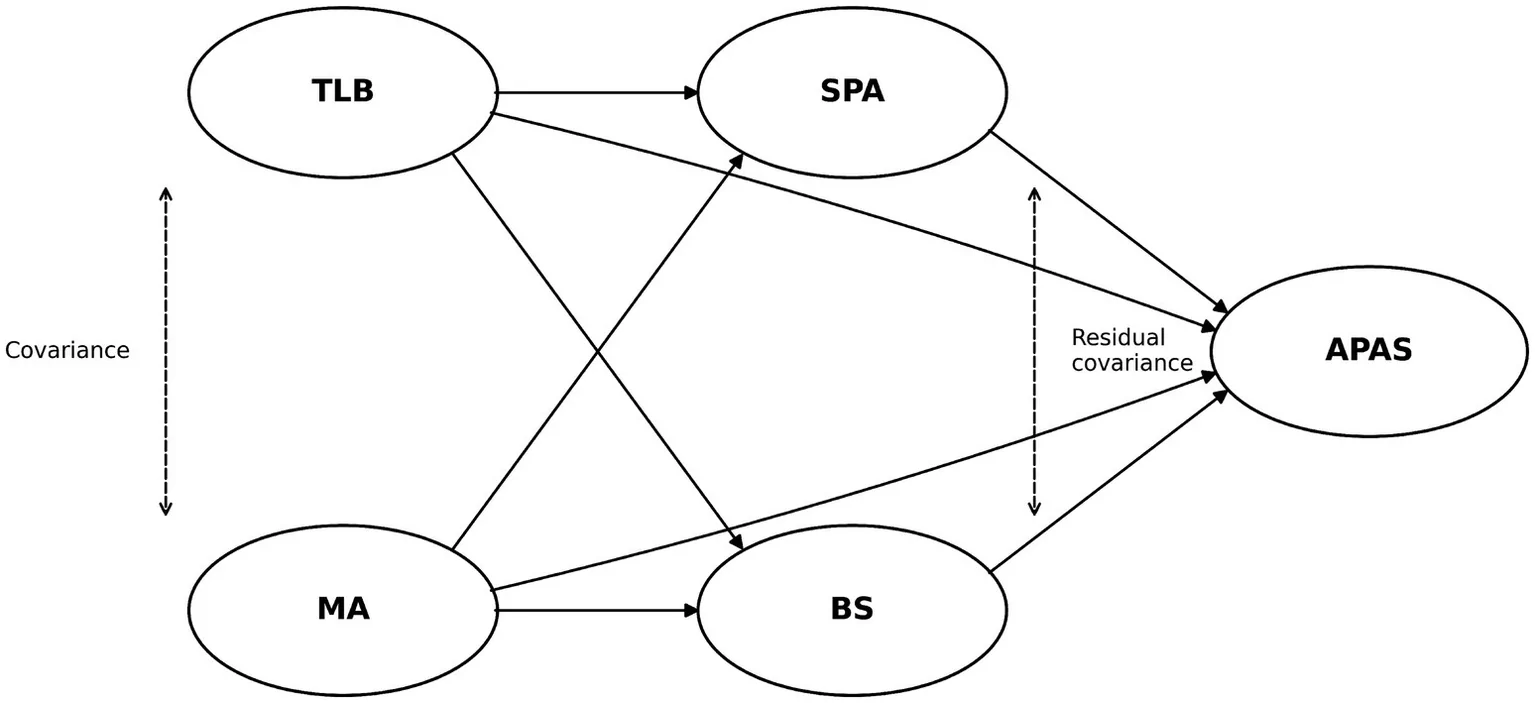
Proposed cross-sectional parallel indirect-association model of appearance-ideal internalization and appearance-related avoidance of physical activity and sport. TLB, thin/low body fat internalization; MA, muscular/athletic internalization; SPA, social physique anxiety; BS, body surveillance; APAS, appearance-related avoidance of physical activity and sport. TLB and MA are specified as correlated predictors. SPA and BS are parallel intermediate variables whose residuals are allowed to covary. Direct paths from TLB and MA to APAS are retained. Directed arrows represent hypothesized cross-sectional associations, not causal effects.

Two directionally specified serial alternatives were examined exploratorily: BS to SPA and SPA to BS. A superior or significant cross-sectional serial specification would still not demonstrate temporal precedence. Equivalent fit would indicate that the observed covariance structure cannot distinguish the ordering. Figure 1 presents the primary conceptual model.

## 3 Methods

### 3.1 Participants and procedure

A cross-sectional online questionnaire was administered between February 20 and March 20, 2026. Participants formed a convenience sample recruited through university-based class groups, student-organization groups, and peer networks at 16 universities in Sichuan Province, China. The final sample included undergraduate and postgraduate students. Eligibility was based on self-report: participants had to be at least 18 years old, currently enrolled at a university, identify their gender as female, read the information statement, and provide electronic written informed consent.

The survey was hosted on Wenjuanxing, and all questionnaire items required a response before submission; the retained analytic dataset therefore contained no missing values. A total of 1,800 questionnaires were returned. Before substantive analysis, submissions completed in less than 180 s were excluded because this duration was considered insufficient for attentive completion of the consent material, background questions, and 35 scale items. The surviving screening record preserved the 180-s rule and three final mutually exclusive categories: 61 submissions below the time threshold, 37 classified during data cleaning as extensive straight-line or patterned responding across scale items, and 15 classified as eligibility inconsistency, possible duplication, or another clearly careless response pattern. It did not preserve the item-level thresholds used to define straight-line or patterned responding, the exact combinations of eligibility responses treated as inconsistent, the platform fields used to flag possible duplicates, preliminary overlap flags, or the decision rules for the residual careless-response category. The 37 and 15 cases therefore represent final classifications recorded during data cleaning rather than categories that can now be independently reproduced from a documented algorithm. Technical screening fields were removed before analysis. The three final categories summed to 113. The final analytic sample comprised 1,687 participants, corresponding to a valid-questionnaire retention proportion of 93.72%. A response rate could not be calculated because the number of eligible students who received or accessed the invitation was unknown.

Participants had a mean age of 20.38 years (SD = 1.41). The 16 university identifiers were retained, with 81 to 127 participants per institution. Class and recruitment-network identifiers were not retained. No *a priori* power analysis was conducted. The achieved sample was large relative to the 35 observed indicators and 115 free parameters in the final latent-variable model and yielded narrow bootstrap confidence intervals for the indirect associations; this description concerns the precision of the achieved sample and is not a retrospective power calculation.

The protocol was approved by the Ethics Committee of the Civil Aviation Flight University of China (approval number 20260051). No names, student identification numbers, telephone numbers, or precise addresses were collected.

### 3.2 Measures

All core constructs were assessed with Chinese-language self-report instruments using five-point response formats. Items were averaged within each scale or subscale after the specified reverse scoring, and higher scores indicated higher levels of the corresponding construct.

#### 3.2.1 Appearance-ideal internalization

The Chinese SATAQ-4 was used to assess TLB and MA (Ma et al., 2023; Schaefer et al., 2015). Each subscale contains five items scored from 1 (strongly disagree) to 5 (strongly agree). Items were averaged separately within the TLB subscale and within the MA subscale, yielding two distinct predictor scores. The two subscales were not combined into a single total score.

#### 3.2.2 Social physique anxiety

SPA was assessed with the seven-item Chinese adaptation evaluated by Jin and Fung (2021). That study compared established and alternative configurations and proposed a Chinese SPAS-7 comprising original SPAS items 2, 4, 6, 7, 9, 10, and 12 for Chinese university samples. Responses ranged from 1 (definitely disagree) to 5 (definitely agree). Original item 2 was reverse-scored before the mean was calculated. The configuration was selected because it was the culturally evaluated seven-item version specified in the cited source, rather than the earlier seven-item configuration using original item 3 in place of item 2.

#### 3.2.3 Body surveillance

BS was measured with the eight-item Body Surveillance Subscale of the Chinese revised Objectified Body Consciousness Scale (OBCS; Chen and Jiang, 2007; McKinley and Hyde, 1996). The documented revision procedure involved administering the original OBCS to 432 Chinese university students, conducting exploratory factor analysis in one subsample (*n* = 212), and evaluating reliability and validity in a second subsample (*n* = 220). The resulting 20-item measure retained three subscales—body surveillance, body shame, and control beliefs—and explained 60.25% of the total variance; item loadings ranged from 0.623 to 0.824, test–retest coefficients from 0.764 to 0.802, and internal-consistency coefficients from 0.861 to 0.875. The accessible published report does not describe a separate forward- and back-translation protocol, and no such procedure is claimed here. The validated Chinese-language items of the eight-item body-surveillance subscale were administered without wording modification and were specified as indicators of a single BS factor in the present analyses. Responses ranged from 1 (completely unlike me) to 5 (completely like me). Items 5 and 6 were positively scored and the remaining items were reverse-scored before averaging. English example items are not reproduced because they were not the administered Chinese wording and no independently documented English back-translation formed part of the survey.

#### 3.2.4 Appearance-related avoidance of physical activity and sport

APAS was assessed with the 10-item Chinese TAPAS (Bevan et al., 2022; Saffari et al., 2023). Items were scored from 1 (strongly disagree) to 5 (strongly agree) and averaged. Higher scores indicated a stronger self-reported tendency to avoid physical activity and sport for body-, weight-, or appearance-related reasons. Item 10 was retained. Prior cross-subcultural differential-item-functioning findings motivated closer examination of this item (Fan et al., 2023). Its performance was evaluated through its standardized loading, corrected item-total correlation, reliability if deleted, residual diagnostics, and a nine-item sensitivity model.

#### 3.2.5 Background characteristics

The survey recorded age, grade, academic major, sports-related-major status, urban or rural origin, monthly living expenses, self-perceived weight status, weekly physical activity frequency, average session duration, daily social media use, and frequency of appearance-related social media exposure. Self-perceived weight was coded from 1 (very underweight) to 5 (very overweight); activity frequency, session duration, and appearance-related media exposure were coded as ordered five-category variables. These measures were used descriptively and in targeted sensitivity, subgroup, or concurrent-validity analyses. They were not treated as objective BMI, body composition, or device-measured activity.

### 3.3 Data analysis

Descriptive, reliability, residual-diagnostic, and auxiliary analyses were conducted in IBM SPSS Statistics 29.0 and R 4.5.3. Alternative measurement-model comparisons were estimated with conventional maximum likelihood in IBM SPSS Amos 26.0. The primary five-factor CFA, structural equation models (SEM), measurement-invariance models, and cluster-adjusted models were estimated in Mplus 8.3. All 35 individual items were used as indicators of their respective latent variables. No correlated item residuals were added, and modification indices were used only for diagnosis.

Item distributions were inspected before latent-variable analysis. Across the 35 items, absolute skewness was no greater than 0.989 and absolute excess kurtosis was no greater than 0.891. Multivariate normality was not assumed as a prerequisite for the primary analysis; five-point items were treated as approximately continuous, and MLR was selected to provide a scaled model test and robust standard errors. A WLSMV model treating all items as ordered categorical indicators was added as an ordinal-estimation sensitivity analysis.

The primary five-factor measurement model specified correlated TLB, MA, SPA, BS, and APAS factors. Alternative one-, two-, three-, and four-factor models were estimated with conventional maximum likelihood to assess empirical distinguishability. Harman’s unrotated single-factor procedure was retained only as a limited descriptive diagnostic; it was not used to claim that common method variance had been excluded (Podsakoff et al., 2003). Reliability was evaluated with Cronbach’s alpha and McDonald’s omega, convergent validity with composite reliability (CR) and average variance extracted (AVE), and discriminant validity with the Fornell-Larcker criterion and the heterotrait-monotrait ratio (HTMT) (Fornell and Larcker, 1981; Henseler et al., 2015).

The primary parallel model used robust maximum likelihood (MLR). TLB and MA were allowed to covary. SPA and BS were regressed on TLB and MA, APAS was regressed on TLB, MA, SPA, and BS, and the residuals of SPA and BS were allowed to covary. The model contained no item-level residual correlations. Model fit was interpreted from the combined pattern of chi-square, CFI, TLI, RMSEA with its 90% confidence interval, and SRMR rather than from a single cutoff (Hu and Bentler, 1999). MLR provided robust standard errors and confidence intervals for model parameters. An otherwise identical maximum-likelihood model with 5,000 bootstrap resamples and seed 20,260,804 provided bias-corrected confidence intervals for unstandardized direct, specific indirect, and total associations (Preacher and Hayes, 2008). Unstandardized estimates were used for inference; standardized coefficients were provided for descriptive comparison. Bootstrap contrasts directly compared the BS- and SPA-specific indirect associations for each predictor.

A specification audit compared four models: no predictor or intermediate-variable covariance (P0), predictor covariance only (P1), intermediate-variable residual covariance only (P2), and both covariances (P3). P3 was designated the primary model on theoretical and statistical grounds. Directionally specified BS-to-SPA and SPA-to-BS models were examined as exploratory competitors. Their fit was not interpreted as evidence of temporal order.

Sensitivity analyses examined ordinal WLSMV estimation; deletion of TAPAS item 10; separate adjustment for urban/rural origin and sports-major status, self-perceived weight, self-reported activity frequency and duration, and appearance-related social media exposure; a combined background model; university clustering; and leave-one-university-out estimates. In each targeted covariate model, the relevant background variable or variable set was entered as a predictor of TLB, MA, SPA, BS, and APAS; the eight primary structural paths, the TLB-MA covariance, and the SPA-BS residual covariance were otherwise retained. Urban/rural and three-group self-perceived-weight comparisons proceeded from configural to metric and scalar invariance before structural-path equality was tested, with changes in fit indices considered alongside robust chi-square difference tests (Chen, 2007). Sports-major comparisons were interpreted cautiously because the sports-major group included 92 participants; an observed-composite model with HC3 standard errors and 5,000 bootstrap resamples was used when the latent multigroup model produced unreliable-standard-error warnings. University clustering was evaluated with intraclass correlations (ICCs), design effects, MLR with TYPE = COMPLEX, and 16 leave-one-university-out models. With only 16 university clusters, cluster-adjusted *p* values were treated as sensitivity evidence rather than decisive inference.

Concurrent associations of APAS with ordered self-reported activity frequency and session duration were examined using Spearman correlations with bootstrap confidence intervals and proportional-odds models adjusted for urban/rural origin, sports-major status, and self-perceived weight. Age was reported descriptively and was not added mechanically to these targeted models.

## 4 Results

### 4.1 Participant characteristics

The analytic sample included 1,687 female university students, including 1,630 undergraduates and 57 postgraduates. Urban and rural origins were nearly balanced. Ninety-two participants were enrolled in a physical education or sports-related major. Slightly more than half described their weight as normal, 21.22% as underweight to some degree, and 26.85% as overweight to some degree. Table 1 presents the full sample characteristics.

**Table 1:** Demographic and background characteristics of participants.

Variable	Category	*n*	%
Age, years	M ± SD	20.38 ± 1.41	—
Grade level	Freshman	456	27.03
	Sophomore	495	29.34
	Junior	397	23.53
	Senior	282	16.72
	Postgraduate	57	3.38
Academic major type	Humanities and social sciences	305	18.08
	Science and engineering	392	23.24
	Medicine and health sciences	241	14.29
	Economics and management	334	19.80
	Arts	167	9.90
	Physical education or sports-related major	92	5.45
	Other	156	9.25
Sports-related major	Yes	92	5.45
	No	1,595	94.55
Place of origin	Urban	816	48.37
	Rural	871	51.63
Monthly living expenses	Less than 1,000 RMB	137	8.12
	1,000–1,999 RMB	672	39.83
	2,000–2,999 RMB	611	36.22
	3,000–3,999 RMB	198	11.74
	4,000 RMB or above	69	4.09
Self-perceived weight status	Very underweight	61	3.62
	Slightly underweight	297	17.61
	Normal weight	876	51.93
	Slightly overweight	382	22.64
	Very overweight	71	4.21
Weekly physical activity frequency	Never	265	15.71
	1–2 times per week	737	43.69
	3–4 times per week	443	26.26
	5–6 times per week	164	9.72
	7 times or more per week	78	4.62
Average duration of each physical activity session	Less than 15 min	259	15.35
	15–29 min	476	28.22
	30–59 min	640	37.94
	60–89 min	222	13.16
	90 min or more	90	5.33
Daily social media use	Less than 1 h	81	4.80
	1–2 h	327	19.38
	2–3 h	514	30.47
	3–4 h	466	27.62
	More than 4 h	299	17.72
Frequency of appearance-related social media exposure	Never	75	4.45
	Rarely	271	16.06
	Sometimes	597	35.39
	Often	521	30.88
	Very often	223	13.22

*N* = 1,687. Percentages may not sum to exactly 100.00 because of rounding. Age is reported as mean ± standard deviation. RMB, Chinese yuan. Self-perceived weight, activity frequency, activity duration, and media exposure were self-reported.

### 4.2 Measurement model, reliability, and discriminant validity

The unrotated principal-components analysis extracted five components with eigenvalues greater than 1, accounting for 64.48% of the total variance; the first component accounted for 32.94%. This result was treated only as a descriptive diagnostic because Harman’s procedure cannot rule out common method variance.

The five-factor model fit substantially better than the alternative combined-factor models, χ^2^(550) = 630.350, CFI = 0.998, TLI = 0.997, RMSEA = 0.009, and SRMR = 0.017. Table 2 presents the conventional-ML alternative-model comparison. This comparison supports empirical distinguishability of the five constructs; it does not demonstrate that same-source method variance is absent. In the independently estimated Mplus five-factor model, the RMSEA 90% CI was [0.005, 0.013].

**Table 2:** Comparison of alternative measurement models.

Model	Specification	χ^2^	df	χ^2^/df	CFI	TLI	RMSEA	SRMR
1	One factor: all indicators	17,031.514	560	30.413	0.516	0.486	0.132	0.131
2	Two factors: TLB + MA; SPA + BS + APAS	13,650.993	559	24.420	0.615	0.590	0.118	0.121
3	Three factors: TLB + MA; SPA + BS; APAS	7,367.114	557	13.226	0.800	0.786	0.085	0.091
4A	Four factors: TLB + MA; SPA; BS; APAS	2,663.411	554	4.808	0.938	0.933	0.048	0.043
4B	Four factors: TLB; MA; SPA + BS; APAS	5,335.946	554	9.632	0.859	0.849	0.072	0.083
4C	Four factors: TLB; MA; SPA; BS + APAS	7,185.645	554	12.970	0.805	0.791	0.084	0.095
5	Five factors: TLB; MA; SPA; BS; APAS	630.350	550	1.146	0.998	0.997	0.009	0.017

Alternative models were estimated with conventional maximum likelihood in Amos 26. A plus sign indicates constructs combined on one factor; semicolons separate distinct factors.

Internal consistency and convergent validity were satisfactory. Alpha and omega ranged from 0.853 to 0.936, standardized loadings ranged from 0.712 to 0.841, CR ranged from 0.854 to 0.936, and AVE ranged from 0.540 to 0.607 (Table 3). All HTMT estimates were below 0.54, and the largest upper bootstrap confidence limit was below 0.58. The square root of AVE for each construct exceeded its correlations with the other constructs (Tables 4, 5). Complete item loadings and residual diagnostics are reported in Supplementary Table S1. The largest absolute residual correlation linking an APAS item with an SPA or BS item was 0.061, below 0.10; empirical separation was supported, while conceptual proximity among appearance-focused items remained possible.

**Table 3:** Descriptive statistics and measurement properties.

Variable	Mean	SD	α	ω	Loading range	CR	AVE
TLB	3.601	0.953	0.883	0.884	0.751–0.841	0.884	0.605
MA	3.597	0.883	0.853	0.854	0.714–0.755	0.854	0.540
SPA	3.430	0.943	0.900	0.900	0.731–0.777	0.900	0.562
BS	3.322	0.965	0.925	0.925	0.712–0.805	0.925	0.607
APAS	3.621	0.929	0.935	0.936	0.735–0.837	0.936	0.594

Loadings are standardized estimates from the five-factor measurement model.

**Table 4:** Correlations and Fornell–Larcker results.

Variable	TLB	MA	SPA	BS	APAS
TLB	**0.778**				
MA	0.468	**0.735**			
SPA	0.312	0.301	**0.750**		
BS	0.375	0.341	0.377	**0.779**	
APAS	0.399	0.371	0.372	0.401	**0.771**

Diagonal values in bold are square roots of AVE. Off-diagonal values are Pearson correlations. All correlations were significant at *p* < 0.01.

**Table 5:** HTMT estimates with bootstrap confidence intervals.

Construct pair	HTMT	95% bootstrap CI
TLB-MA	0.539	[0.495, 0.579]
TLB-SPA	0.349	[0.297, 0.400]
MA-SPA	0.343	[0.291, 0.395]
TLB-BS	0.413	[0.367, 0.459]
MA-BS	0.383	[0.334, 0.430]
SPA-BS	0.413	[0.370, 0.457]
TLB-APAS	0.439	[0.392, 0.484]
MA-APAS	0.415	[0.369, 0.460]
SPA-APAS	0.406	[0.361, 0.450]
BS-APAS	0.431	[0.388, 0.472]

### 4.3 Primary parallel structural model

The specification audit showed that the model omitting both the TLB-MA covariance and the SPA-BS residual covariance yielded SRMR = 0.093. Allowing only the predictor covariance reduced SRMR to 0.043, whereas allowing only the intermediate-variable residual covariance reduced SRMR to 0.078. The theoretically complete P3 model, which estimated both covariances without adding item-residual correlations, showed the strongest fit. The primary MLR results were robust χ^2^(550) = 600.111, CFI = 0.998, TLI = 0.998, RMSEA = 0.007, 90% CI [0.000, 0.011], and SRMR = 0.017. Because the latent-variable structural portion of P3 was saturated, these global indices reflected the adequacy of the five-factor measurement structure and did not independently validate the direction of the structural paths. The full P0–P3 audit is reported in Supplementary Table S2.

**Figure 2:**
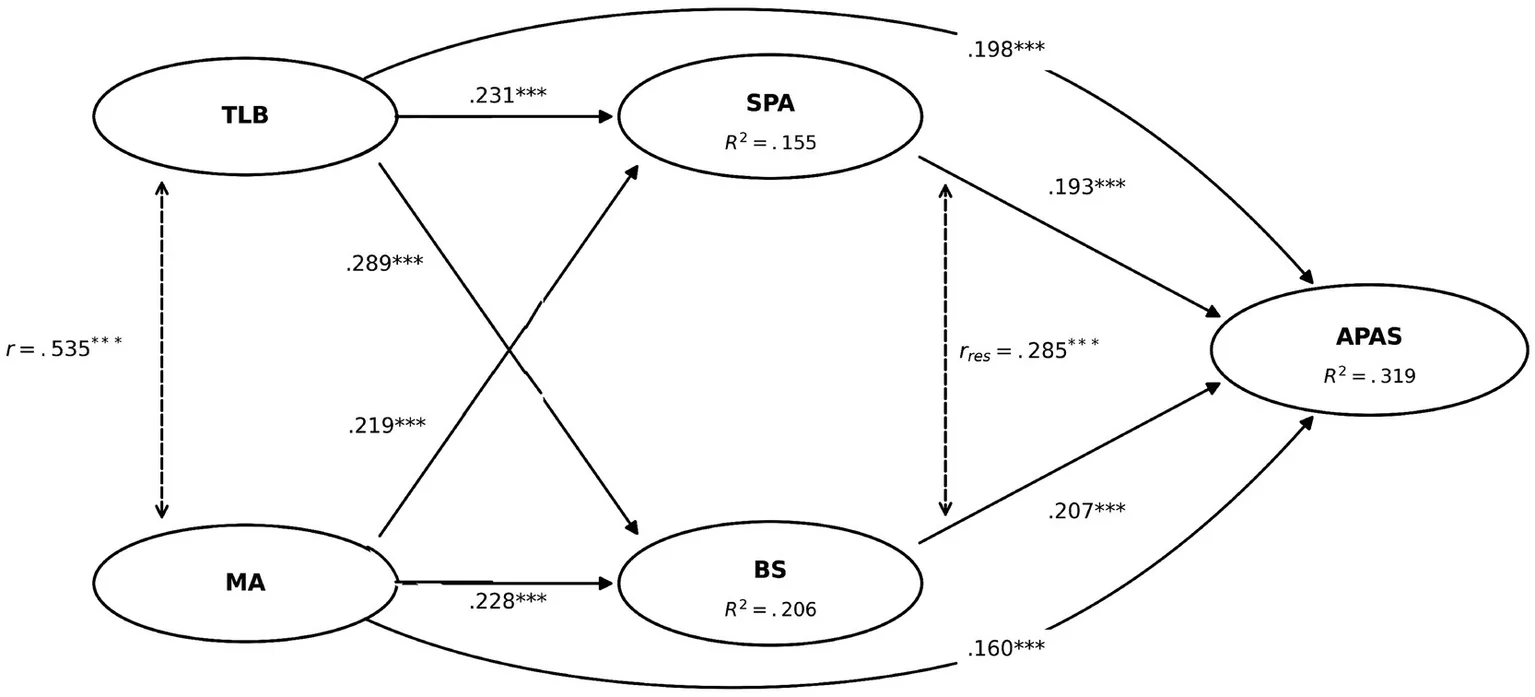
Standardized primary parallel model of appearance-ideal internalization and APAS. Values on single-headed arrows are STDYX coefficients. The dashed double-headed arrow between TLB and MA shows their standardized covariance. The dashed double-headed arrow between SPA and BS shows their standardized residual covariance. Values inside endogenous variables are R-squared. All displayed estimates were significant at *p* < 0.001.

TLB and MA were positively correlated, standardized *r* = 0.535, *p* < 0.001. After their associations with TLB and MA were modeled, the residual correlation between SPA and BS was 0.285, *p* < 0.001. All eight specified direct paths were positive and statistically significant (Table 6). TLB and MA each showed positive conditional direct associations with APAS, supporting H1a and H1b. TLB and MA were also positively associated with SPA and BS, and both SPA and BS were positively associated with APAS. The model explained 15.5% of the variance in SPA, 20.6% in BS, and 31.9% in APAS (see Figure 2).

**Table 6:** Primary structural path estimates.

Path	B	Robust SE	z	*p*	95% CI for B	Standardized *β*
TLB → SPA	0.207	0.029	7.098	<0.001	[0.150, 0.264]	0.231
MA → SPA	0.245	0.038	6.506	<0.001	[0.171, 0.319]	0.219
TLB → BS	0.280	0.030	9.202	<0.001	[0.221, 0.340]	0.289
MA → BS	0.275	0.038	7.176	<0.001	[0.200, 0.351]	0.228
TLB → APAS	0.188	0.029	6.394	<0.001	[0.130, 0.245]	0.198
MA → APAS	0.190	0.036	5.244	<0.001	[0.119, 0.261]	0.160
SPA → APAS	0.205	0.029	7.155	<0.001	[0.149, 0.261]	0.193
BS → APAS	0.203	0.028	7.333	<0.001	[0.149, 0.257]	0.207

B, robust SE, z, *p*, and confidence intervals are unstandardized MLR results. Standardized beta values are STDYX estimates.

### 4.4 Direct, total, and specific parallel indirect associations

All four prespecified specific indirect associations were statistically significant (Table 7). TLB showed specific indirect associations with APAS involving SPA, B = 0.042, 95% bias-corrected CI [0.028, 0.061], and BS, B = 0.057, CI [0.039, 0.077], supporting H2a and H3a. MA showed specific indirect associations involving SPA, B = 0.050, CI [0.032, 0.073], and BS, B = 0.056, CI [0.037, 0.079], supporting H2b and H3b. The direct associations remained statistically significant after SPA and BS were included; this indicates that the estimated specific indirect associations did not account for the entire cross-sectional association.

**Table 7:** Direct, total, and specific parallel indirect associations.

Predictor	Association	B	Bootstrap SE	95% bias-corrected CI	Standardized estimate
TLB	Direct: TLB → APAS	0.188	0.029	[0.128, 0.245]	0.198
	Specific indirect: TLB → SPA → APAS	0.042	0.008	[0.028, 0.061]	0.045
	Specific indirect: TLB → BS → APAS	0.057	0.010	[0.039, 0.077]	0.060
	Total indirect	0.099	0.013	[0.076, 0.125]	0.104
	Total association	0.287	0.030	[0.229, 0.346]	0.302
MA	Direct: MA → APAS	0.190	0.036	[0.121, 0.262]	0.160
	Specific indirect: MA → SPA → APAS	0.050	0.010	[0.032, 0.073]	0.042
	Specific indirect: MA → BS → APAS	0.056	0.011	[0.037, 0.079]	0.047
	Total indirect	0.106	0.015	[0.079, 0.138]	0.089
	Total association	0.296	0.037	[0.225, 0.369]	0.249

Unstandardized estimates, bootstrap standard errors, and confidence intervals were obtained from an ML model with 5,000 bootstrap resamples. Standardized estimates are provided for descriptive comparison. The table does not report proportions of a causal process.

The difference between the BS- and SPA-specific indirect associations was not statistically supported for TLB, B = 0.015, 95% bias-corrected CI [−0.012, 0.039], *p* = 0.272, or MA, B = 0.006, CI [−0.024, 0.036], *p* = 0.706. The data therefore supported the presence of both specific indirect associations when estimated simultaneously, without evidence that one was larger than the other.

### 4.5 Sensitivity, subgroup, and concurrent-activity analyses

The principal paths and specific indirect associations were stable across the targeted sensitivity analyses (Table 8; Supplementary Tables S4–S10). Adjusting separately for urban/rural origin and sports-major status, self-perceived weight, activity frequency and duration, or appearance-related social media exposure did not change the direction or inferential conclusion of any primary path or specific indirect association. The largest absolute standardized-path change was 0.033 in the media-exposure model and 0.013 in the activity model; changes in the other covariate models were 0.001 or smaller.

**Table 8:** Summary of sensitivity and boundary-condition analyses.

Analysis	Key evidence	Interpretation
Background covariates	All eight paths and four specific indirect associations retained their direction and CI conclusion; maximum absolute standardized change = 0.033	Principal pattern was stable across targeted covariate models
Urban/rural origin	Structural equality: robust Δχ^2^(8) = 10.262, *p* = 0.247	No detectable omnibus path difference
Self-perceived weight	Structural equality: robust Δχ^2^(16) = 12.348, *p* = 0.720	No detectable omnibus path difference across three perceived-weight groups
Sports-related major	Absolute Hedges g ≤ 0.088; bootstrap group-difference CIs included zero	No detectable difference, with limited precision for n = 92
University clustering	ICC range 0.000–0.007; maximum design effect = 1.78; leave-one-university-out estimates stable	Results were not driven by a single university; only 16 clusters were available
TAPAS item 10	Loading = 0.742; corrected item-total *r* = 0.715; maximum path change after deletion = 0.004	Primary results did not depend on item 10
WLSMV estimation	CFI = 0.999, TLI = 0.999, RMSEA = 0.006, SRMR = 0.016; maximum path difference = 0.012	Conclusions were stable when items were treated as ordinal
Concurrent activity	Frequency ρ = −0.243; duration ρ = −0.112	APAS was related to, but distinct from, self-reported activity behavior

Urban/rural metric and scalar invariance were supported, and constraining all eight structural paths to equality did not significantly worsen fit, robust Δχ^2^(8) = 10.262, *p* = 0.247. For the self-perceived-weight groups (underweight *n* = 358, normal weight n = 876, overweight *n* = 453), metric and scalar invariance were also supported, and the omnibus structural-path equality test was not significant, robust Δχ^2^(16) = 12.348, *p* = 0.720. These results did not identify group differences in the specified paths; they do not establish complete equivalence.

The sports-major group was small (*n* = 92) relative to the non-sports-major group (*n* = 1,595). Mean differences were negligible across the five constructs, absolute Hedges g ≤ 0.088, all *p* > 0.40. The latent multigroup model produced an unreliable-standard-error warning, so inferential emphasis was placed on observed-composite comparisons with HC3 standard errors and 5,000 bootstrap resamples. No bootstrap confidence interval for a group difference excluded zero. These results were treated as exploratory and imprecise.

University-level ICCs ranged from 0.000 to 0.007. The largest design effect was 1.78 for TLB. TYPE = COMPLEX estimates preserved all path coefficients and inferential conclusions, although only 16 clusters were available. Across 16 leave-one-university-out models, all path directions remained positive and standardized shifts were small. University-cluster findings were therefore used as sensitivity evidence rather than definitive multilevel inference.

TAPAS item 10 had a standardized loading of 0.742 and a corrected item-total correlation of 0.715. Alpha and omega would each have remained 0.930 if the item had been deleted. Removing item 10 changed standardized structural paths by no more than 0.004. The 10-item scale was retained. Treating all items as ordered categorical indicators with WLSMV also produced closely similar results; standardized-path differences from MLR were no larger than 0.012, and every primary path remained significant at *p* < 0.001.

APAS was concurrently associated with lower self-reported weekly activity frequency, Spearman ρ = −0.243, 95% bootstrap CI [−0.289, −0.196], and shorter self-reported session duration, ρ = −0.112, CI [−0.161, −0.066]. In proportional-odds models adjusted for urban/rural origin, sports-major status, and self-perceived weight, a one-standard-deviation higher APAS score was associated with lower odds of being in a higher activity-frequency category, OR = 0.616, 95% CI [0.562, 0.675], and lower odds of being in a longer-duration category, OR = 0.811, 95% CI [0.744, 0.885]. These are concurrent associations based on self-report, not objective activity or causal effects. Full results are reported in Supplementary Table S11.

### 4.6 Exploratory serial alternatives

The BS-to-SPA and SPA-to-BS alternatives had identical robust fit to the primary parallel model: robust χ^2^(550) = 600.111, CFI = 0.998, TLI = 0.998, RMSEA = 0.007, SRMR = 0.017, AIC = 151,968.289, BIC = 152,592.820, and log likelihood = −75,869.144. Complete serial products were statistically different from zero in both parameterizations, but the models were statistically equivalent re-expressions of the same cross-sectional covariance structure. They did not identify whether BS preceded SPA, SPA preceded BS, or reciprocal relations were present. The parallel model remained the primary, more assumption-limited representation. Full results and diagrams are provided in Supplementary Table S3 and Supplementary Figures S1, S2.

## 5 Discussion

### 5.1 Interpretation of the main findings

Both appearance-ideal internalization dimensions showed positive conditional direct associations with APAS. The TLB result is consistent with the possibility that physical activity becomes uncomfortable when it highlights body size, body fat, or deviation from a thin ideal. The MA result requires a more qualified interpretation. Athletic and fitness ideals can motivate movement, exercise, or appearance-based self-improvement (Homan and Tylka, 2014), yet they can also intensify discrepancy, comparison, or dissatisfaction when a person evaluates whether the body looks sufficiently toned or athletic (Homan, 2010; Robinson et al., 2017). A higher APAS score does not show that MA corresponds to less physical activity overall. It indicates that stronger MA can coexist with a stronger tendency to avoid particular body-evaluative situations. The negative concurrent associations between APAS and self-reported activity frequency and duration support behavioral relevance, but their modest magnitude also reinforces the distinction between an avoidance tendency and actual participation.

SPA and BS each retained a positive association with APAS when estimated simultaneously. SPA captures apprehension about external evaluation. Exercise may heighten this apprehension through public movement, clothing, performance, or comparison. BS captures habitual attention to how the body appears from an observer’s perspective. That attentional orientation can shift experience away from enjoyment, competence, or bodily function (Cox et al., 2019; Gilchrist et al., 2021). The four specific indirect associations were statistically supported, but the cross-sectional data cannot establish a psychological sequence. Reverse and reciprocal patterns remain plausible: repeated avoidance of body-evaluative settings could reinforce anxiety, monitoring, or sensitivity to appearance ideals.

The bootstrap contrasts did not support larger indirect associations involving BS than SPA, or vice versa. The theoretical contribution therefore lies in retaining two conceptually distinct correlates within the same model, not in ranking their importance. Their residual correlation also indicates contemporaneous overlap after TLB and MA are considered. Measurement-model results showed that SPA, BS, and APAS were empirically distinguishable, while small residual correlations do not eliminate conceptual proximity among items concerning observation, judgment, or appearance during activity.

### 5.2 Cultural context and theoretical implications

The findings add culturally situated evidence from female university students attending universities in Sichuan Province. Chinese appearance culture reflects both local and transnational influences. Young Chinese women may encounter thinness, low-body-fat, toned, and athletic ideals in domestic social media, commercial fitness content, peer discussion, and campus movement settings. Prospective evidence suggests that Asian appearance media can be more salient than Western appearance media for some body-image concerns among young Chinese women (Jackson et al., 2020). Chinese social-media research also documents body-positive efforts that resist appearance anxiety while retaining substantial appearance-focused content (Lang et al., 2023; Lang and Ye, 2024). The current data cannot determine whether the magnitudes of the observed associations differ from those in Western samples, and no cross-cultural claim is warranted without matched samples and measurement-invariance testing.

Three contributions are supported. The model distinguishes thin/low body fat from muscular/athletic internalization, avoiding the reduction of contemporary female appearance standards to thinness alone. It focuses on the specific TAPAS-defined avoidance tendency rather than general inactivity. It also estimates SPA and BS simultaneously, preserving the distinction between interpersonal-evaluative anxiety and intrapersonal body monitoring. These contributions are bounded by the correlational design and by the finding that directionally specified serial models were statistically equivalent.

### 5.3 Practical implications

The findings identify candidate components for future intervention trials; no intervention was tested. For students with strong TLB, weight-stigma-free and non-weight-focused fitness options could be evaluated. Candidate elements include avoiding public weigh-ins, body-fat ranking, transformation challenges, and praise centered on weight loss or visible size change. Course messaging could emphasize enjoyment, health, comfort, stress regulation, and sustainable participation.

For students with strong MA, competence-oriented sport workshops could be evaluated. Skill acquisition, movement quality, functional strength, personal progress, and teamwork can be emphasized without treating a toned or athletic-looking body as evidence of competence. Instructors can avoid appearance comparison, physique commentary, and visual transformation targets that equate athleticism with a single body shape.

SPA-related components could reduce unnecessary evaluative threat through beginner-friendly instruction, flexible participation formats, private feedback, and explicit norms against commenting on bodies. BS-related components could direct attention toward breathing, balance, coordination, energy, strength, or other aspects of bodily function, consistent with body-functionality approaches (Alleva et al., 2015). Media-literacy components could address image editing, filters, commercial motives, algorithmic repetition, and the overlap between thinness and fitness imagery. Research on social media influencer marketing highlights digital engagement and communication between influencers and followers, including the promotion of brands and products through social media platforms (Fauzi et al., 2024). The survey measured general appearance-related social-media exposure rather than a dedicated short-video variable, so short-video-specific recommendations remain proposals for future evaluation.

### 5.4 Limitations and future research

The cross-sectional design precludes temporal and causal conclusions. The serial alternatives demonstrate the problem directly: BS-to-SPA, SPA-to-BS, and parallel specifications fit identically. Longitudinal designs with at least four appropriately spaced assessments could separate internalization, BS, SPA, and APAS across waves and test reciprocal effects. Even longitudinal ordering would not by itself establish causality; experimental or intervention designs would be needed for stronger causal inference where feasible.

All core variables were measured at the same time by self-report. Social desirability, recall, shared response context, and common method variance may have inflated associations. Harman’s procedure and alternative substantive factor models cannot statistically exclude common method variance. Conceptual proximity is also possible because TAPAS, SPA, and BS all refer in different ways to appearance, observation, or evaluation. The five-factor model, loadings, HTMT values, and residual diagnostics support empirical distinguishability, but they do not prove completely separate psychological processes.

The convenience sample was recruited through online university networks in one province. Participation was voluntary, the invitation denominator was unavailable, and a response rate could not be calculated. The de-identified analytic dataset retained university codes but not class or recruitment-network identifiers. This permitted ICC, TYPE = COMPLEX, and leave-one-university-out sensitivity analyses at the university level; however, 16 clusters were insufficient for a complex university-level latent model, and cluster-adjusted standard errors were interpreted cautiously. The original 1,800-record screening file, participant-level flags, item-level thresholds for straight-line or patterned responding, exact eligibility-response combinations, platform fields used for duplicate checks, and preliminary overlap indicators were not retained with the analytic dataset. Consequently, the 37 and 15 cases represent archived final cleaning classifications rather than independently reproducible algorithmic categories. The loss of the original screening audit limits independent reconstruction of the 113 exclusions. Together with voluntary recruitment and the unknown invitation denominator, these features constrain representativeness and precision.

Objective height, weight, BMI, body composition, device-measured activity, and verified physical education attendance were not collected. Self-perceived weight and self-reported activity frequency and duration provided useful sensitivity and concurrent-validity evidence, yet they cannot substitute for objective measures. Future studies could combine TAPAS with researcher-measured anthropometrics, seven-day accelerometry, class-attendance records, gym-entry records, and ecological momentary reports of avoidance in specific settings.

A stronger sampling design would use stratified multistage recruitment across eastern, central, western, and northeastern China, include comprehensive, normal, sports, and vocational institutions, retain school and class identifiers, and use multilevel SEM. Matched cross-national samples would require configural, metric, and scalar invariance before structural differences were interpreted. Oversampling sports-major students would improve precision for that subgroup. Dedicated measures of short-video exposure, appearance-focused algorithmic content, weight stigma, perceived competence, and prior negative exercise experiences could clarify boundary conditions.

## 6 Conclusion

In this convenience sample of 1,687 female university students recruited from 16 universities in Sichuan Province, TLB and MA each showed positive conditional direct associations with APAS. SPA and BS each retained positive associations with APAS and statistically significant specific parallel indirect associations involving both internalization dimensions. The pattern remained stable across multiple sensitivity analyses, and APAS was concurrently associated with lower self-reported activity frequency and shorter duration. The cross-sectional findings do not establish temporal ordering, causal mechanisms, cross-cultural differences, or intervention effectiveness.

## Data Availability

The original contributions presented in the study are included in the article/Supplementary material, further inquiries can be directed to the corresponding author.
