# Supplementary material for: Appearance-ideal internalization and appearance-related physical activity avoidance among female university students in Sichuan, China: the role of social physique anxiety and body surveillance
Source: Front Psychol. 2026 Sep 8;17:1870099. doi: 10.3389/fpsyg.2026.1870099 (PMC13593480; doi:10.3389/fpsyg.2026.1870099)
Supplement: Supplementary file 1 [file Supplementary_file_1.DOCX]

# **Supplementary Material**

**Manuscript ID:** 1870099

**Title:** *Appearance-Ideal Internalization and Appearance-Related Physical Activity Avoidance Among Female University Students in Sichuan, China: The Role of Social Physique Anxiety and Body Surveillance*

**Abbreviations:** APAS = appearance-related avoidance of physical activity and sport; BS = body surveillance; MA = muscular/athletic internalization; SPA = social physique anxiety; TLB = thin/low body fat internalization.

# **Supplementary Table S1. Item Loadings and Measurement Diagnostics**

## **Table S1A. Standardized Factor Loadings**

| **Construct** | **Item** | **Loading** | **SE** | **p** |
| --- | --- | --- | --- | --- |
| TLB | X1_1 | 0.841 | 0.009 | <.001 |
| TLB | X1_2 | 0.78 | 0.011 | <.001 |
| TLB | X1_3 | 0.751 | 0.012 | <.001 |
| TLB | X1_4 | 0.754 | 0.012 | <.001 |
| TLB | X1_5 | 0.758 | 0.012 | <.001 |
| MA | X2_1 | 0.716 | 0.014 | <.001 |
| MA | X2_2 | 0.749 | 0.013 | <.001 |
| MA | X2_3 | 0.739 | 0.013 | <.001 |
| MA | X2_4 | 0.714 | 0.014 | <.001 |
| MA | X2_5 | 0.755 | 0.013 | <.001 |
| SPA | M1_1 | 0.746 | 0.012 | <.001 |
| SPA | M1_2 | 0.741 | 0.013 | <.001 |
| SPA | M1_3 | 0.756 | 0.012 | <.001 |
| SPA | M1_4 | 0.731 | 0.013 | <.001 |
| SPA | M1_5 | 0.763 | 0.012 | <.001 |
| SPA | M1_6 | 0.733 | 0.013 | <.001 |
| SPA | M1_7 | 0.777 | 0.011 | <.001 |
| BS | M2_1 | 0.805 | 0.01 | <.001 |
| BS | M2_2 | 0.778 | 0.011 | <.001 |
| BS | M2_3 | 0.712 | 0.013 | <.001 |
| BS | M2_4 | 0.793 | 0.01 | <.001 |
| BS | M2_5 | 0.8 | 0.01 | <.001 |
| BS | M2_6 | 0.787 | 0.01 | <.001 |
| BS | M2_7 | 0.788 | 0.01 | <.001 |
| BS | M2_8 | 0.768 | 0.011 | <.001 |
| APAS | Y1 | 0.837 | 0.008 | <.001 |
| APAS | Y2 | 0.735 | 0.012 | <.001 |
| APAS | Y3 | 0.76 | 0.011 | <.001 |
| APAS | Y4 | 0.796 | 0.01 | <.001 |
| APAS | Y5 | 0.772 | 0.011 | <.001 |
| APAS | Y6 | 0.737 | 0.012 | <.001 |
| APAS | Y7 | 0.8 | 0.01 | <.001 |
| APAS | Y8 | 0.758 | 0.011 | <.001 |
| APAS | Y9 | 0.762 | 0.011 | <.001 |
| APAS | Y10 | 0.742 | 0.012 | <.001 |

*Note.* All loadings are STDYX estimates from the five-factor model. No correlated item residuals were added. The largest absolute residual correlation linking an APAS item with an SPA or BS item was .061.

## **Table S1B. HTMT Estimates and Bootstrap Confidence Intervals**

| **Pair** | **HTMT** | **95% CI** |
| --- | --- | --- |
| TLB-MA | 0.539 | [0.495, 0.579] |
| TLB-SPA | 0.349 | [0.297, 0.400] |
| MA-SPA | 0.343 | [0.291, 0.395] |
| TLB-BS | 0.413 | [0.367, 0.459] |
| MA-BS | 0.383 | [0.334, 0.430] |
| SPA-BS | 0.413 | [0.370, 0.457] |
| TLB-APAS | 0.439 | [0.392, 0.484] |
| MA-APAS | 0.415 | [0.369, 0.460] |
| SPA-APAS | 0.406 | [0.361, 0.450] |
| BS-APAS | 0.431 | [0.388, 0.472] |

# **Supplementary Table S2. Parallel-Model Specification Audit**

| **Model** | **Specification** | **χ²** | **df** | **CFI** | **TLI** | **RMSEA (90% CI)** | **SRMR** | **AIC** | **BIC** |
| --- | --- | --- | --- | --- | --- | --- | --- | --- | --- |
| P0 | No predictor covariance; no SPA-BS residual covariance | 1151.96 | 552 | 0.982 | 0.981 | 0.025 [0.023, 0.027] | 0.093 | 152,485.899 | 153,099.569 |
| P1 | TLB-MA covariance only | 740.787 | 551 | 0.994 | 0.994 | 0.014 [0.011, 0.017] | 0.043 | 152,076.727 | 152,695.827 |
| P2 | SPA-BS residual covariance only | 1042.2 | 551 | 0.986 | 0.984 | 0.023 [0.021, 0.025] | 0.078 | 152,378.135 | 152,997.236 |
| P3 | Both covariances | 630.349 | 550 | 0.998 | 0.997 | 0.009 [0.005, 0.013] | 0.017 | 151,968.289 | 152,592.820 |

Note. Maximum-likelihood values are shown to provide a common specification audit. P3 was retained as the primary model with MLR inference. The P0 SRMR was .093.

# **Supplementary Table S3. Indirect-Association Contrasts and Exploratory Serial Alternatives**

## **Table S3A. Bootstrap Contrasts Between Specific Indirect Associations**

| **Contrast** | **B** | **Bootstrap SE** | **p** | **95% bias-corrected CI** |
| --- | --- | --- | --- | --- |
| TLB: BS-specific minus SPA-specific | 0.015 | 0.013 | 0.272 | [-0.012, 0.039] |
| MA: BS-specific minus SPA-specific | 0.006 | 0.015 | 0.706 | [-0.024, 0.036] |

## **Table S3B. Fit of the Parallel and Directionally Specified Serial Models**

| **Model** | **Robust χ²** | **df** | **CFI** | **TLI** | **RMSEA** | **SRMR** | **AIC** | **BIC** | **Log likelihood** |
| --- | --- | --- | --- | --- | --- | --- | --- | --- | --- |
| Parallel P3 | 600.111 | 550 | 0.998 | 0.998 | 0.007 | 0.017 | 151,968.289 | 152,592.820 | -75,869.144 |
| Serial alternative S1: BS → SPA | 600.111 | 550 | 0.998 | 0.998 | 0.007 | 0.017 | 151,968.289 | 152,592.820 | -75,869.144 |
| Serial alternative S2: SPA → BS | 600.111 | 550 | 0.998 | 0.998 | 0.007 | 0.017 | 151,968.289 | 152,592.820 | -75,869.144 |

## **Table S3C. Complete Serial Products**

| **Direction** | **Predictor** | **B** | **Bootstrap SE** | **p** | **95% bias-corrected CI** |
| --- | --- | --- | --- | --- | --- |
| BS → SPA | TLB | 0.016 | 0.003 | <.001 | [0.010, 0.023] |
| BS → SPA | MA | 0.015 | 0.003 | <.001 | [0.010, 0.024] |
| SPA → BS | TLB | 0.013 | 0.003 | <.001 | [0.008, 0.019] |
| SPA → BS | MA | 0.015 | 0.003 | <.001 | [0.009, 0.022] |

*Note.* The three models had identical fit and likelihood. Statistical significance of a serial product in an equivalent cross-sectional parameterization does not establish temporal order.

**Supplementary Figure S1. Directionally Specified Serial Alternative With Body Surveillance Associated With Social Physique Anxiety**

Note. TLB and MA were allowed to covary. The BS-to-SPA direction replaced the residual covariance between BS and SPA, while all direct paths to APAS were retained. This exploratory cross-sectional competitor does not establish temporal ordering. TLB = thin/low body fat internalization; MA = muscular/athletic internalization; SPA = social physique anxiety; BS = body surveillance; APAS = appearance-related avoidance of physical activity and sport.

**Supplementary Figure S2. Directionally Specified Serial Alternative With Social Physique Anxiety Associated With Body Surveillance**

Note. TLB and MA were allowed to covary. The SPA-to-BS direction replaced the residual covariance between SPA and BS, while all direct paths to APAS were retained. This exploratory cross-sectional competitor does not establish temporal ordering. TLB = thin/low body fat internalization; MA = muscular/athletic internalization; SPA = social physique anxiety; BS = body surveillance; APAS = appearance-related avoidance of physical activity and sport.

# **Supplementary Table S4. Targeted Covariate Sensitivity Analyses**

| **Sensitivity model** | **Maximum absolute change in standardized path** | **All directions retained** | **All eight paths p < .05** | **CI conclusion retained** |
| --- | --- | --- | --- | --- |
| Urban/rural origin and sports-major status | 0.001 | Yes | Yes | Yes |
| Five-level self-perceived weight status | 0.001 | Yes | Yes | Yes |
| Self-reported activity frequency and duration | 0.013 | Yes | Yes | Yes |
| Appearance-related social-media exposure | 0.033 | Yes | Yes | Yes |
| Combined urban/rural, sports-major, and perceived-weight model | 0.001 | Yes | Yes | Yes |
| Combined background model with university clustering | 0.001 | Yes | Yes | Yes |

*Note. Four specific indirect associations also retained their direction and confidence-interval conclusion in every model. Background variables were examined in separate theory-based models rather than entered as one generic control set. In each model, the listed covariate or covariate set was entered as a predictor of TLB, MA, SPA, BS, and APAS; the eight primary structural paths, the TLB-MA covariance, and the SPA-BS residual covariance were otherwise retained.*

# **Supplementary Table S5. Urban-Rural Measurement Invariance and Structural Comparison**

| **Comparison** | **Robust Δχ²** | **df** | **p** | **ΔCFI** | **ΔRMSEA** | **ΔSRMR** |
| --- | --- | --- | --- | --- | --- | --- |
| Metric vs. configural | 25.675 | 30 | 0.692 | 0 | 0 | 0.002 |
| Scalar vs. metric | 31.042 | 30 | 0.413 | 0 | 0 | 0 |
| Equal vs. freely estimated structural paths | 10.262 | 8 | 0.247 | 0 | 0 | 0.001 |

*Note.* Urban n = 816; rural n = 871. No path-specific comparison remained significant after Benjamini-Hochberg adjustment. Failure to detect differences is not proof of complete equality.

# **Supplementary Table S6. Self-Perceived Weight Measurement Invariance and Structural Comparison**

| **Comparison** | **Robust Δχ²** | **df** | **p** | **ΔCFI** | **ΔRMSEA** | **ΔSRMR** |
| --- | --- | --- | --- | --- | --- | --- |
| Metric vs. configural | 59.518 | 60 | 0.493 | 0 | 0 | 0.003 |
| Scalar vs. metric | 59.317 | 60 | 0.501 | 0 | -0.001 | 0.001 |
| Equal vs. freely estimated structural paths | 12.348 | 16 | 0.72 | 0 | -0.001 | 0.002 |

*Note.* Underweight n = 358, normal weight n = 876, and overweight n = 453. Self-perceived weight is not BMI or objectively measured weight. No path-specific comparison remained significant after Benjamini-Hochberg adjustment.

# **Supplementary Table S7. Sports-Major Sensitivity Analyses**

## **Table S7A. Composite-Score Group Descriptives**

| **Construct** | **Sports major M (SD)** | **Non-sports M (SD)** | **Hedges g** | **p** |
| --- | --- | --- | --- | --- |
| TLB | 3.541 (1.022) | 3.605 (0.950) | -0.066 | 0.563 |
| MA | 3.524 (0.888) | 3.601 (0.883) | -0.088 | 0.418 |
| SPA | 3.421 (0.974) | 3.430 (0.941) | -0.01 | 0.926 |
| BS | 3.269 (1.007) | 3.325 (0.963) | -0.058 | 0.604 |
| APAS | 3.579 (0.915) | 3.624 (0.930) | -0.048 | 0.653 |

*Note.* Sports major n = 92; non-sports major n = 1,595. The latent configural model generated an unreliable-standard-error warning. Observed-composite HC3 and 5,000-resample bootstrap comparisons were therefore used as the inferential fallback. All bootstrap confidence intervals for sports-minus-non-sports path differences included zero.

# **Supplementary Table S8. University Counts, ICCs, and Cluster Sensitivity**

## **Table S8A. Participants per University**

| **University code** | **n** | **%** |
| --- | --- | --- |
| 1 | 113 | 6.7 |
| 2 | 103 | 6.11 |
| 3 | 109 | 6.46 |
| 4 | 122 | 7.23 |
| 5 | 81 | 4.8 |
| 6 | 104 | 6.16 |
| 7 | 111 | 6.58 |
| 8 | 98 | 5.81 |
| 9 | 127 | 7.53 |
| 10 | 117 | 6.94 |
| 11 | 88 | 5.22 |
| 12 | 112 | 6.64 |
| 13 | 110 | 6.52 |
| 14 | 105 | 6.22 |
| 15 | 90 | 5.33 |
| 16 | 97 | 5.75 |

## **Table S8B. ICCs and Design Effects**

| **Construct** | **ICC** | **Equal-size design effect** | **Unequal-size design effect** |
| --- | --- | --- | --- |
| TLB | 0.0074 | 1.772 | 1.783 |
| MA | 0.003 | 1.317 | 1.321 |
| SPA | 0 | 1 | 1 |
| BS | 0 | 1 | 1 |
| APAS | 0 | 1 | 1 |

## **Table S8C. Ordinary and TYPE = COMPLEX Path Estimates**

| **Path** | **B ordinary** | **SE ordinary** | **B complex** | **SE complex** | **Standardized beta** |
| --- | --- | --- | --- | --- | --- |
| BS→APAS | 0.203 | 0.028 | 0.203 | 0.02 | 0.207 |
| MA→APAS | 0.19 | 0.036 | 0.19 | 0.041 | 0.16 |
| MA→BS | 0.275 | 0.038 | 0.275 | 0.032 | 0.228 |
| MA→SPA | 0.245 | 0.038 | 0.245 | 0.028 | 0.219 |
| SPA→APAS | 0.205 | 0.029 | 0.205 | 0.029 | 0.193 |
| TLB→APAS | 0.188 | 0.029 | 0.188 | 0.019 | 0.198 |
| TLB→BS | 0.28 | 0.03 | 0.28 | 0.023 | 0.289 |
| TLB→SPA | 0.207 | 0.029 | 0.207 | 0.023 | 0.231 |

## **Table S8D. Leave-One-University-Out Stability**

| **Path** | **Range of standardized change** |
| --- | --- |
| BS→APAS | [-0.009, 0.008] |
| MA→APAS | [-0.019, 0.014] |
| MA→BS | [-0.013, 0.012] |
| MA→SPA | [-0.012, 0.011] |
| SPA→APAS | [-0.008, 0.015] |
| TLB→APAS | [-0.008, 0.007] |
| TLB→BS | [-0.010, 0.011] |
| TLB→SPA | [-0.016, 0.009] |

*Note.* All leave-one-university-out paths remained positive and statistically significant. Only 16 clusters were available; TYPE = COMPLEX results are sensitivity evidence, not a substitute for a well-powered multilevel model.

# **Supplementary Table S9. TAPAS Item 10 and Nine-Item Sensitivity**

## **Table S9A. TAPAS Item 10 Diagnostics**

| **Item 10 diagnostic** | **Value** |
| --- | --- |
| Mean | 3.525 |
| SD | 1.161 |
| Skewness | -0.539 |
| Excess kurtosis | -0.329 |
| Corrected item-total correlation | 0.715 |
| Alpha if deleted | 0.93 |
| Omega if deleted | 0.93 |
| Standardized loading | 0.742 |
| Largest absolute residual with SPA/BS item | 0.039 |

## **Table S9B. Structural-Path Comparison**

| **Path** | **APAS10 beta** | **APAS9 beta** | **Change** |
| --- | --- | --- | --- |
| BS→APAS | 0.207 | 0.209 | 0.002 |
| MA→APAS | 0.16 | 0.156 | -0.004 |
| MA→BS | 0.228 | 0.228 | 0 |
| MA→SPA | 0.219 | 0.219 | 0 |
| SPA→APAS | 0.193 | 0.19 | -0.003 |
| TLB→APAS | 0.198 | 0.201 | 0.003 |
| TLB→BS | 0.289 | 0.289 | 0 |
| TLB→SPA | 0.231 | 0.231 | 0 |

*Note.* Item 10 was retained in the primary scale. Deletion was used only as a sensitivity analysis.

# **Supplementary Table S10. MLR and WLSMV Sensitivity**

## **Table S10A. Model-Fit Comparison**

| **Estimator** | **χ²** | **df** | **CFI** | **TLI** | **RMSEA (90% CI)** | **SRMR** |
| --- | --- | --- | --- | --- | --- | --- |
| MLR | 600.111 | 550 | 0.998 | 0.998 | 0.007 [0.000, 0.011] | 0.017 |
| WLSMV | 587.447 | 550 | 0.999 | 0.999 | 0.006 [0.000, 0.010] | 0.016 |

## **Table S10B. Standardized Path Comparison**

| **Path** | **MLR beta** | **WLSMV beta** | **Difference** |
| --- | --- | --- | --- |
| BS→APAS | 0.207 | 0.205 | -0.002 |
| MA→APAS | 0.16 | 0.172 | 0.012 |
| MA→BS | 0.228 | 0.224 | -0.004 |
| MA→SPA | 0.219 | 0.22 | 0.001 |
| SPA→APAS | 0.193 | 0.202 | 0.009 |
| TLB→APAS | 0.198 | 0.188 | -0.01 |
| TLB→BS | 0.289 | 0.29 | 0.001 |
| TLB→SPA | 0.231 | 0.23 | -0.001 |

*Note.* AIC and BIC are not compared across MLR and WLSMV. All primary paths were significant at p < .001 under both estimators.

# **Supplementary Table S11. Concurrent Associations With Self-Reported Activity**

## **Table S11A. Spearman Correlations**

| **Activity indicator** | **Spearman ρ** | **95% bootstrap CI** |
| --- | --- | --- |
| Weekly frequency | -0.243 | [-0.289, -0.196] |
| Session duration | -0.112 | [-0.161, -0.066] |

## **Table S11B. Adjusted Proportional-Odds Models**

| **Outcome** | **B** | **SE** | **z** | **p** | **OR** | **95% CI for OR** |
| --- | --- | --- | --- | --- | --- | --- |
| Higher activity-frequency category | -0.484 | 0.047 | -10.417 | <.001 | 0.616 | [0.562, 0.675] |
| Longer session-duration category | -0.209 | 0.044 | -4.72 | <.001 | 0.811 | [0.744, 0.885] |

*Note.* Predictor = standardized APAS. Models adjusted for urban/rural origin, sports-major status, and self-perceived weight. Frequency and duration were self-reported ordered categories; the estimates are concurrent, not causal.
